# Correlation of Serum Prolactin and Thyroid Stimulating Hormone Concentration in Infertile Women: A Systematic Review and Meta-Analysis

**DOI:** 10.21315/mjms2024.31.1.2

**Published:** 2024-02-28

**Authors:** Delini Devi Ramadras, Noor Azlin Azraini Che Soh@Yusof, Najib Majdi Yaacob, Wan Azman Wan Norlina, Abdul Hamid Hanisah

**Affiliations:** 1 Department of Chemical Pathology, School of Medical Sciences, Universiti Sains Malaysia, Kelantan, Malaysia; 2 Unit of Biostatistics and Research Methodology, School of Medical Science, Universiti Sains Malaysia, Kelantan, Malaysia; 3 Chemical Pathology Unit, Department of Pathology, Hospital Tengku Ampuan Rahimah, Selangor, Malaysia

**Keywords:** thyroid stimulating hormone, prolactin, infertility, correlation, meta-analysis

## Abstract

Infertility affects millions of people of reproductive age worldwide. Thyroid hormones and prolactin (PRL) affect reproduction and pregnancy; therefore, these two hormones influence fertility. This systematic review and meta-analysis aimed to summarise the strength of the correlation between serum PRL and thyroid stimulating hormone (TSH) in infertile women and to explore selected factors influencing the correlation. We conducted a systematic search of online databases (PubMed, Scopus, ScienceDirect, SAGE and Google Scholar) from inception until March 2021 and a manual search of the bibliographies of the included studies to identify relevant publications. The original research paper describing the correlation between PRL and TSH in reproductive-age women with infertility (primary and secondary) was included. The risk of bias was assessed using the Joanna Briggs Institute Critical Appraisal Checklist for Analytical Cross-Sectional Studies. A random effect model was used to estimate the pooled correlations of PRL and TSH, followed by an assessment of heterogeneity and a sensitivity analysis. From a total of 822 relevant articles identified, 11 were eligible and included in this systematic review and meta-analysis. The random effect pooled correlation estimates between PRL and TSH was 0.431 (95% CI: 0.251, 0.582), with substantial heterogeneity between the included studies (*I*^2^ = 80%, *τ*^2^ = 0.067, *P* < 0.001). No significant publication bias was observed. Study region, types of infertility, sample size and year of the study did not influence the correlation estimates. Our results highlighted a significant positive moderate correlation between serum PRL and serum TSH in infertile women.

## Introduction

Serum prolactin (PRL) and thyroid stimulating hormone (TSH) play a significant role in a couple’s fecundity. These hormones are regulated by the same pathway. The elevated thyrotrophin-releasing hormone level in hypothyroidism increases PRL secretion (1). Hypothyroidism and hyperprolactinaemia are found to be closely interrelated (2).

Serum TSH is elevated when stimulated by the increased production of TRH. This increment, along with follicle-stimulating hormone (FSH) and luteinising hormone (LH), has synergistic action at the granulosa cell level. This stimulation affects the growth and secretion of steroid hormones from the ovaries, which are responsible for normal reproductive function, thus causing infertility (3, 4).

There is no negative feedback on TRH because thyroid hormone production is low in hypothyroidism, thus allowing excessive PRL release (5). Elevated PRL levels potentially affect fertility by suppressing the hypothalamic-pituitary-gonadal axis and gonadotropin-releasing hormone (GnRH) pulsatility. This occurs when elevated PRL levels interfere with the secretion and action of gonadotrophins in growing follicles in the ovaries, thus impairing gonadal steroid secretion, which further affects positive feedback to gonadotropins. This leads to follicle immaturity and consequently infertility with anovulation (4).

In addition, a decrement in sex hormone-binding globulin production occurs, as hypothyroidism also alters the peripheral metabolism of oestrogen. Potentially, this could be another pathway via which there may result abnormal feedback at the pituitary level, thus impairing fertility (6). Furthermore, hyperprolactinaemia can contribute to infertility due to elevated levels of dopamine affecting steroidogenesis as a result of GnRH inhibition (7).

Infertility associated with elevated PRL and TSH levels is reversible with treatment, irrespective of the type of treatment. Therefore, assessing serum PRL and TSH is essential in determining the cause of infertility. We believe the correlation between serum PRL and TSH in infertile women would carry a good weightage to the general population, particularly concerning infertility management.

## Methods

### Design and Protocol Development

The systematic review and meta-analysis were conducted and reported according to the Preferred Reporting Items for Systematic Reviews and Meta-Analyses (PRISMA) (8). A protocol was registered in the International Prospective Register of Systematic Reviews database (PROSPERO 2021 CRD42021249901) (Appendix 1).

### Eligibility Criteria

The following inclusion criteria were applied: original studies; the inclusion of populations of women of reproductive age with infertility (primary and secondary); and reporting of the correlation between PRL and TSH. Studies including male factor infertility and female factors such as tubal factors, any congenital abnormalities of the urogenital tract or any apparent organic lesions were excluded. Studies including patients with a history of thyroid disease, thyroid surgery or thyroid medication use were also excluded. Experimental (randomised, non-randomised) trials, ecological studies, case reports, studies not involving human participants (animal and in-vitro studies), book chapters, narrative reviews and study protocols were also excluded.

### Data Sources and Search Strategy

A systematic search of multiple databases using the search terms listed above was conducted to identify articles published in peer-reviewed journals, clinical trial registries and conference proceedings. Two investigators (DDR and NAACS) extensively searched the MEDLINE (PubMed), Scopus, ScienceDirect, SAGE and Google Scholar databases from inception up to March 2021. Bibliographies of included studies were hand-searched to identify additional relevant studies. To maximise the sensitivity of the literature search, we included all MESH terms for PRL, TSH and infertility using the OR Boolean operator to connect all the terms (Appendix 2). Forward and backward reference chaining of included studies was carried out in which the reference lists from the included papers were searched to identify other relevant information.

### Selection Process

Two review authors (DDR and NAACS) conducted preliminary screening of titles and abstracts to identify potentially eligible studies. The full text of these studies was then retrieved and assessed for eligibility criteria. Assessment of eligibility was conducted in duplicate and independently to avoid bias in study selection. The degree of change-adjusted agreement between both review authors was noted. Conflicts in study identification were discussed and resolved in conjunction with a third review author (WNWA). All review authors had to reach complete agreement. Detailed assessments of the reasons for exclusion were documented.

### Data Extraction

Data extraction from eligible studies into a standardised pre-designed electronic data extraction form in Microsoft Excel format was performed by two independent review authors (DDR and NAACS). The following variables were extracted: author’s name, publication year, country, region, study design, study population (age group and type of infertility), method of measuring serum PRL and TSH, number of study participants and strength of correlation (r) between PRL and TSH. The data were analysed following the resolution of overlaps in the extracted data. When there were multiple publications of the same study, data were extracted from each publication, but only the most complete and up-to-date data were included. The literature search and screening output were reported in a PRISMA study flow diagram.

### Methodological Quality Assessment

The quality of each included study (assessment of bias) was critically and objectively appraised by two reviewers (DDR and NAACS) independently and in duplicate using the Joanna Briggs Institute (JBI) Critical Appraisal Checklist for Observational Studies (9). This checklist consists of eight questions for cross-sectional studies. The questions assess specific domains of studies to determine the potential risk of bias and can be answered with ‘yes’, ‘no’, ‘unclear’ or ‘not applicable’. Any disagreements between reviewers were reviewed and resolved by discussion with the involvement of a third reviewer (WNWA). All reviewers agreed upon decisions about the scoring system and cut-off points before critical appraisal commenced, as recommended by the JBI reviewers’ manual (10). Studies were judged according to the following: low risk of bias if 70% of answers scored a ‘yes’, moderate risk if 50%–69% of the questions scored a ‘yes’ and high risk of bias if ‘yes’ scores were below 50%.

### Data Synthesis and Statistical Analysis

The descriptions of original studies were summarised using tables and forest plots. Data were entered in a Microsoft Excel file before performing statistical analysis using meta package (11) in the RStudio environment (version 1.4.1717) of R version 4.0.3 (12). Fisher’s *z*-transformation of the correlations was carried out before pooling the correlation estimates using the inverse variance method. Substantial variability is expected in the designs of individual studies (differing population, biochemical method), which will result in substantial variability in the actual effect size. Therefore, we consider a random effect model the most appropriate method to compute the summary effect size. The random effect model with Hartung-Knapp adjustment was therefore used to estimate the pooled correlation and a 95% confidence interval (CI) was computed.

### Heterogeneity Assessment

Forest plots, the tau-squared (τ^2^) statistic, the Higgins I squared (*I*^2^) statistic and the *P*-value of a Cochran’s Q test were used to determine heterogeneity among the included studies (13). The τ^2^ statistic was obtained using the Hunter-Schmidt estimator, while the Q-profile method was used to obtain the CI of the τ^2^ statistic. In our meta-analysis, the τ^2^ statistic is the between-study variance. It estimates the variance of the underlying distribution of true effect sizes (14). The Higgins *I*^2^ statistic indicates low heterogeneity when the value is less than 25%, moderate heterogeneity when the value is between 25% and 75% and substantial heterogeneity when the value is 75% or higher (15). Statistical heterogeneity was also evaluated using the *P*-value of a Cochran’s Q test in which *P* < 0.10 indicates the presence of statistically significant heterogeneity.

### Subgroup and Sensitivity Analysis

Subgroup analyses were conducted to explore possible causes of heterogeneity. The random effects model with separate estimates was applied for all categorical moderator variables. We also performed a robustness analysis to assess the impact of each study on the pooled result by removing one at a time from the analysis for all studies (leave-one-out analysis).

## Results

### Study Selection

**Figure 1:**
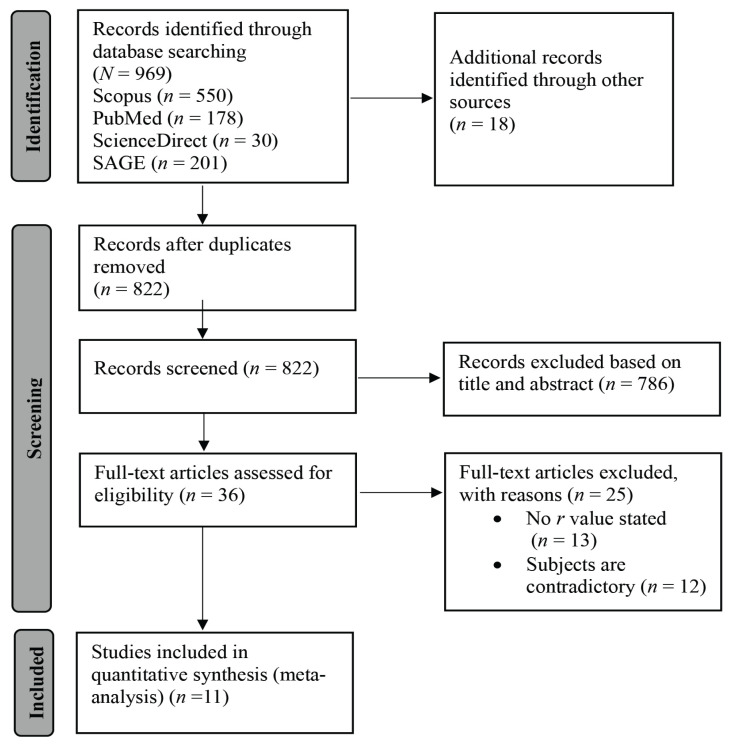
PRISMA flowchart: a meta-analysis of the correlation between serum PRL and serum TSH in infertile women

After eliminating the duplicates, 822 articles were screened, of which 36 were found to be related to our study. By reviewing the full text of these 36 articles and considering the predetermined inclusion criteria, 11 articles (two cross-sectional studies and nine case-control studies) with valuable and related data for meta-analysis were selected (Figure 1). A total of 760 patients were investigated in our systematic review and meta-analysis.

### Study Characteristics

The characteristics of the included studies are described in Table 1 (4, 7, 16–24). The studies were published between 2009 and 2019. Nine of the studies are case control studies and the remaining two are cross-sectional studies. Considerable heterogeneity between studies was observed due to the different populations and methods used. Eight studies were conducted in South Asia, four in the Eastern Mediterranean region and one in the European region. Sample sizes ranged from 40 to 160 subjects of reproductive age.

The methods used in the studies to detect PRL and TSH levels were primarily enzyme-linked immunosorbent assays (ELISAs), followed by electrochemiluminescence immunoassays (ECLIAs), chemiluminescence immunoassays (CLIAs), enzyme immunoassays (EIAs) and enzyme-linked fluorescent assays (ELFAs). Of the studies, four involved subjects with primary infertility, two involved subjects with secondary infertility and seven involved subjects with primary and secondary infertility. There were two articles which have stratified their patients into primary and secondary infertility (17, 23).

### Risk of Bias

All the included studies were classified as having a low risk of bias according to the JBI Critical Appraisal Checklist for Cross-Sectional Studies (Table 2). This indicates that the studies included are of high quality.

### Correlation of Serum PRL and TSH in Infertile Women

**Figure 2:**
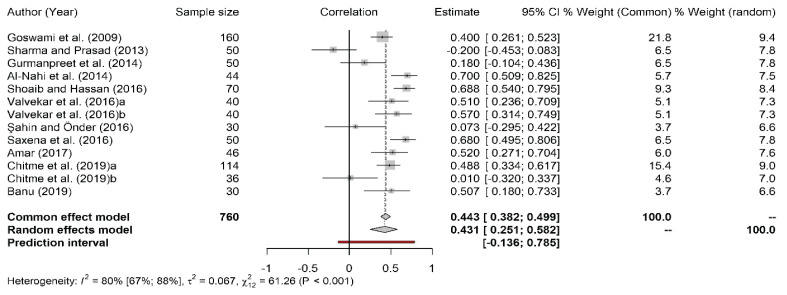
Forest plot of the meta-analysis for the correlation of serum PRL and TSH in infertile women

Figure 2 presents the overall outcome of this study involving all 11 articles comprising a total of 760 subjects. The correlation between serum PRL and TSH in infertile women was measured as 0.431 (95% CI: 0.251, 0.582) for the random effect model against a *P*-value of < 0.001. We found that almost all studies had a positive correlation between serum PRL and TSH in infertile women, except for the study by Sharma and Prasad (18), which shows a negative correlation. Heterogeneity (*I*^2^) was 80%, which is substantial.

### Subgroup Analysis

Variation in the correlation between serum PRL and TSH according to study region was explored by grouping the studies according to World Health Organization (WHO) member state regions (South Asia region and Eastern Mediterranean region). A study from the European region was excluded because it was the only study from that region; subgroup analysis was not applicable. The highest random effects pooled correlation observed in the Eastern Mediterranean region was about 0.522. *I*^2^ observed in the South Asia and Eastern Mediterranean regions was 79% and 84%, respectively.

Further exploration of the variation in the correlation was carried out according to the type of infertility. The summary estimates and heterogeneity are summarised in Table 3. The type of infertility was classified as either primary only, secondary only or primary and secondary. Of the 11 studies, four involved subjects with primary infertility, two involved subjects with secondary infertility and the remainder involved subjects with primary and secondary infertility. *I*^2^ was observed among subjects with secondary infertility and subjects with primary and secondary infertility (86% and 87%, respectively). Moderate heterogeneity (*I*^2^ = 36%) was found among the subjects with primary infertility.

A statistically significant between-group difference was detected when the subgroup analysis was conducted according to type of infertility, with a *P*-value of 0.195 for primary infertility. A lower correlation estimate was observed among studies involving only subjects with secondary infertility compared to studies involving only subjects with primary infertility (0.322 versus 0.413).

### Sensitivity Analysis

**Figure 3:**
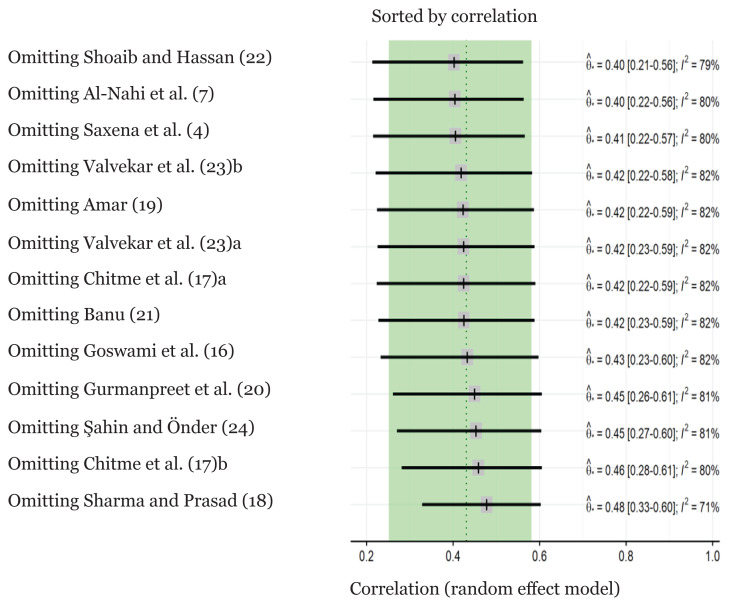
Sensitivity analysis sorted by correlation

A sensitivity analysis was conducted by excluding one study (Figure 3). The effect of excluding the outlier was that there was no significant alteration in combined correlation and 95% CI values, indicating the high stability of this meta-analysis.

### Quality Assessment and Publication Bias

**Figure 4:**
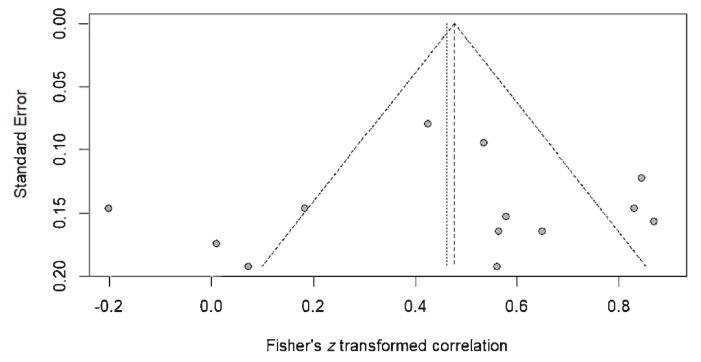
Funnel plot of publication bias

A Begg rank correlation test and an Egger’s test for small-study effects indicated there was no publication bias among all the included studies. This is evident because *z* < 1.96 (*z* = −0.80) and *P* > 0.05 (*P* = 0.423) in the Begg’s test, and *P* > 0.05 (*P* = 0.725) in the Egger’s test. The funnel plot symmetry is in agreement with the results of the Egger’s test (Figure 4).

## Discussion

This meta-analysis showed a positive correlation between serum PRL and TSH in infertile women, with a pooled correlation estimate of 0.431. This means an increase in serum PRL is accompanied by an increase in serum TSH and vice-versa. This is because hyperprolactinaemia and hypothyroidism (which are characterised by high levels of serum TSH and low thyroxine) are closely connected. In women of reproductive age, hypothyroidism can prevent consistent ovulation. It has been shown that some women with high PRL levels had hypothyroidism (25).

However, among the studies included in this meta-analysis, only the one by Sharma and Prasad (18) was negatively correlated. The author postulates that the study populations hailing from the Sub-Himalayan Goitre Belt may have contributed to this negative correlation because of the short feedback loop exemplified by the pituitary hormone (TSH in this case), which has negative feedback on the hypothalamus via a cognate receptor. A significant aspect of the hormonal cascade is that the negative feedback loop kicks in when the ultimate hormone level have been released into the bloodstream at sufficiently high quantities (18). This unfavourable feedback indirectly lowers PRL levels.

Variation in the pooled correlation estimate was observed when the subgroup analysis was conducted according to region. Regarding the Eastern Mediterranean region, the subgroup analysis indicates a higher pooled effect estimate compared to South Asia. This may be because one of the studies conducted in the South Asia region had a negative correlation (18). This subgroup analysis needs to be interpreted with caution due to the small number of studies from the Eastern Mediterranean region (*n* = 4) and the South Asian region (*n* = 8). As the difference in effect sizes across the subgroups narrows, the number of studies required for subgroup analysis grows exponentially (26). In this subgroup meta-analysis, we could not compare the effect estimate for the European region, as there was only one study carried out in this region.

The subgroup analysis of the subject groups with primary, secondary and both primary and secondary infertility revealed a significant difference. A statistically significant between-group difference was detected with a *P*-value of 0.195 for the subjects with primary infertility. Testing for within-group heterogeneity revealed that the subject group with primary infertility had moderate heterogeneity (36%) compared to the subject groups with secondary infertility and both primary and secondary infertility (86% and 87%, respectively). Infertility in women is a very complex condition that can vary for different reasons, including genetic, environmental, social, nutritional, endocrine, neurological and family reasons (27). The impact of other influencing elements must therefore be considered. Primary infertility most frequently affects women aged 20 years old–45 years old, with a peak prevalence between the ages of 25 years old and 35 years old. However, the incidence of secondary infertility is not uniform in terms of age (28). This and the above factors may have resulted in the difference in heterogeneity.

### Study Significance

In this meta-analysis, serum PRL and TSH in infertile women appeared well correlated, as evidenced by the closely linked hypothalamic-pituitary-thyroid axis and the hypothalamic-pituitary-ovarian axis (29). Notably, it can be stated that hypothyroidism influences ovarian function by increasing the secretion of PRL and decreasing the levels of sex hormone-binding globulin. Elevated PRL levels inhibit FSH and GnRH, two hormones necessary for ovulation (30). FSH and LH do not induce the synthesis of gonadal steroid hormones when GnRH secretion is low because FSH and LH are also low (31, 32). This continuous interaction between elevated PRL and TSH has a significant effect on women’s infertility. Nevertheless, it is crucial to note that infertility due to elevated serum PRL and TSH is reversible with prompt diagnosis and treatment (1).

## Conclusion

To the best of our knowledge, this is the first meta-analysis examining the correlation of serum PRL and TSH in infertile women. The pooled correlation of serum PRL and TSH was 0.431 among infertile women, indicating that the finding of hyperprolactinaemia is common among patients with hypothyroidism. As a significant correlation exists between serum PRL and TSH, all infertile women should be evaluated for both serum PRL and TSH.

## Limitation

The limitations of this study are that not all included articles specify the mean age of the subjects. Therefore, a subgroup analysis of this was not possible. Furthermore, several studies did not specify the exact confounding factors that were prioritised before the blood-taking procedure. It is crucial to note that the majority of the subjects in this meta-analysis were from South Asia and the Mediterranean region and therefore do not represent the global population.

## Figures and Tables

**Table 1:** Correlation between serum PRL and serum TSH in infertile women in the included studies

Author	Year of publication	Country	Design	Age (years old)	Type of fertility	*n*	Method of PRL detection	Method of TSH detection	hPRL *(n*)	Elevated TSH (n)	*r*
Goswami et al. (16)	2009	India	CC	20–40	primary	160	ECLIA	ECLIA	65	13	0.400
Chitme et al. (17)a	2019	Oman	CS	19–45	primary	114	CLIA	ELISA	16	11	0.488
Chitme et al. (17)b	2019	Oman	CS	19–45	secondary	36	CLIA	ELISA	8	2	0.010
Sharma and Prasad (18)	2013	India	CC	20–40	mixed	50	ELISA	ELISA	19	18	0.200
Amar (19)	2017	India	CC	21–42	mixed	46	CLIA	CLIA	ns	ns	0.520
Gurmanpreet et al. (20)	2014	India	CC	ns	primary	50	ELISA	ELISA	ns	ns	0.180
Banu (21)	2019	Nepal	CS	15–29	mixed	30	CLIA	CLIA	17	11	0.507
Shoaib and Hassan (22)	2016	Sudan	CC	20–40	mixed	70	EIA	EIA	17	2	0.688
Valvekar et al. (23)a	2016	India	CC	23–39	primary	40	ELFA	ELFA	36	44	0.510
Valvekar et al. (23)b	2016	India	CC	23–39	secondary	40	ELFA	ELFA	36	44	0.570
Şahin and Önder (24)	2016	Turkey	CC	15–45	mixed	40	EIA	EIA	ns	ns	0.073
Saxena et al. (4)	2016	India	CC	20–40	mixed	50	ELISA	ECLIA	12	10	0.680
Al-Nahi et al. (7)	2014	Iraq	CC	22–35	mixed	44	ELISA	ELISA	13	9	0.700

Notes: CC = case control; CS = cross-sectional; *n* = number of patients; CLIA = chemiluminescence immunoassay; ECLIA = electrochemiluminescence immunoassay; EIA = enzyme immunoassay; ELFA = enzyme-linked fluorescence assay; ELISA = enzyme-linked immunosorbent assay; hPRL = hyperprolactinaemia; TSH = thyroid stimulating hormone; *r* = correlation value; ns = not stated

**Table 2:** Assessed risk of bias in the included studies

Author	Goswami et al.	Chitme et al.	Sharma and Prasad	Amar	Gurmanpreet et al.	Banu	Shoaib and Hassan	Valvekar et al.	Şahin and Önder	Saxena et al.	Al-Nahi et al.
1. Were the criteria for inclusion in the sample clearly defined?	Yes	Yes	Yes	Yes	Yes	Yes	Yes	Yes	Yes	Yes	Yes
2. Were the study subjects and the setting described in detail?	Yes	Yes	Yes	Yes	Yes	Yes	Yes	Yes	Yes	Yes	Yes
3. Was the exposure measured in a valid and reliable way?	Yes	Yes	Yes	Yes	Yes	Yes	Yes	Yes	Yes	Yes	Yes
4. Were objective, standard criteria used to measure the conditions?	Yes	Yes	Yes	Yes	Yes	Yes	Yes	Yes	Yes	Yes	Yes
5. Were confounding factors identified?	Yes	Yes	Yes	Yes	Yes	Yes	Yes	Yes	Yes	Yes	Yes
6. Were strategies to deal with confounding factors stated?	Yes	Yes	Yes	Yes	Yes	Yes	Yes	Yes	Yes	Yes	Yes
7. Were the outcomes measured in a valid and reliable way?	Yes	Yes	Yes	Yes	Yes	Yes	Yes	Yes	Yes	Yes	Yes
8. Was appropriate statistical analysis used?	Yes	Yes	Yes	Yes	Yes	Yes	Yes	Yes	Yes	Yes	Yes
Grading	100%	100%	100%	100%	100%	100%	100%	100%	100%	100%	100%
Risk of bias	Low	Low	Low	Low	Low	Low	Low	Low	Low	Low	Low

**Table 3:** Subgroup analysis of the correlation between serum PRL and TSH in infertile women

Study characteristics	Number of studies	Random effect of pooled correlation	95% CI of pooled correlation	Within-group heterogeneity			Between-group heterogeneity	
				*I*^2^(%)	τ^2^	*P*-value for Q	*χ*^2^ (df)	*P*-value
Region								
South Asia	8	0.414	0.170, 0.611	79	0.0565	< 0.001	3.89 (2)	0.143
Eastern Mediterranean region	4	0.522	0.028, 0.829	84	0.0591	< 0.001		
European region	1	0.073	−0.295, 0.422	–	–	–		
Type of fertility								
Primary	4	0.413	0.214, 0.579	36	0.0020	0.195	0.31 (2)	0.856
Secondary	2	0.322	0, 1.0	86	0.0727	0.008		
Primary and secondary	7	0.472	0.124, 0.717	87	0.1317	< 0.001		

Notes: CI = confidence interval; τ^2^ = tau-squared statistic; I^2^ = Higgins *I* squared statistic; Q = Cochran’s Q; ^2^ = chi-square; df = degrees of freedom

## Appendix 2 MeSH terms

1. Prolactin (https://www.ncbi.nlm.nih.gov/mesh/68011388)
  Mammotropic Hormone, Pituitary
  Hormone, Pituitary Mammotropic
  Pituitary Mammotropic Hormone
  PRL (Prolactin)
  Lactogenic Hormone, Pituitary
  Hormone, Pituitary Lactogenic
  Pituitary Lactogenic Hormone
  Mammotropin
2. Thyroid stimulating hormone (https://www.ncbi.nlm.nih.gov/mesh/68013972)
  TSH (Thyroid Stimulating Hormone)
  Thyrotrophin
  Thyroid-Stimulating Hormone
  Hormone, Thyroid-Stimulating
  Thyroid Stimulating Hormone
  Thyreotropin
3. Infertility (https://www.ncbi.nlm.nih.gov/mesh/68007246)
  Sterility, Reproductive
  Sterility
  Reproductive Sterility
  Subfertility
  Sub-Fertility

Database:

1. PubMed: https://pubmed.ncbi.nlm.nih.gov/
2. Scopus:https://www.scopus.com/search/form.uri?display=basic&zone=header&origin=#basic
3. ScienceDirect:
4. Google Scholar
5. SAGE
